# Ischemic post-conditioning is neuroprotective even at delayed tPA administration after embolic stroke in female rats

**DOI:** 10.22038/IJBMS.2021.55674.12456

**Published:** 2021-12

**Authors:** Mohadeseh Mohammadi, Masoud Mobini, Fatemeh Mashayekhi, Mohammad Allahtavakoli, Ayat Kaeidi, Jalal Hassanshahi, Ali Shamsizadeh, Elham Hakimizadeh, Mahsa Hassanipour

**Affiliations:** 1 Student Research Committee, Rafsanjan University of Medical Sciences, Rafsanjan, Iran; 2 Department of Nursing, School of Nursing and Midwifery, Jiroft University of Medical Sciences, Jiroft, Iran; 3 Physiology-Pharmacology Research Center, Research Institute of Basic Medical Sciences, Rafsanjan University of Medical Sciences, Rafsanjan, Iran; 4 Department of Physiology and Pharmacology, School of Medicine, Rafsanjan University of Medical Sciences, Rafsanjan, Iran

**Keywords:** Embolic stroke, Female rat, Ischemic post-conditioning, Neuroprotection, Tissue plasminogen - activator

## Abstract

**Objective(s):**

Delayed tissue plasminogen activator (tPA) thrombolysis is accompanied by different complications in stroke patients. Studies reported sex differences in stroke therapy. Ischemic postconditioning (PC) unveils neuroprotection in stroke models. In this study, we investigate the combined effect of delayed tPA therapy and PC procedure during an embolic stroke experimental model in female rats.

**Materials and Methods:**

Female Wistar rats were randomly divided into control (saline), tPA, PC, and tPA+PC groups after stroke induction via clot injection to the middle cerebral artery. tPA treatment was initiated 6 hr after stroke, and PC procedure was performed 6.5 hr post-ischemia induction (occlusion: 10 sec; reopening: 30 sec; 5 cycles). The cerebral blood flow (CBF) was recorded up to 60 min from IV tPA injection time. The parameters of brain edema, infarct volume, disruption of the blood-brain barrier (BBB), behavioral tests, and matrix metalloproteinases-9 (MMP-9) were evaluated.

**Results:**

This study revealed that PC conduction prevents excessive CBF increase by tPA and played a protective role in infarct volume reduction (*P*<0.05). The combination of PC and tPA reduced the infarct volume, brain edema, and protected BBB. tPA+PC could alleviate neurobehavioral disorders compared with control or tPA. Moreover, PC had the capability of MMP-9 reduction when combined with delayed tPA (*P*<0.05).

**Conclusion:**

Conduction of PC not only alleviated some stroke complications but also enhanced the therapeutic time window of tPA in female rats under embolic stroke.

## Introduction

Stroke, an episode of neurological deficit, is associated with a focal acute injury to the central nervous system (CNS) (1). The incidence of stroke, pathologic mechanisms, and treatment efficacy have been influenced by the gender factor, which reinforces the development of gender-specific therapy (2). The stroke incidence is lower in pre-menopausal women in comparison with men, attributed to the anti-inflammatory and neuroprotective effects of gonadal steroids (estrogen) (3), and it is recognized as a major health problem in elderly postmenopausal women (4). Furthermore, by the evidence exhibiting that men and women experience stroke in a different manner, gender must be taken into account when treating the stroke patients and also when designing experimental studies or clinical trials (5).

Inflammation has a fundamental role in stroke (6), and data revealed that higher inflammatory responses of ischemic stroke in old animals are accompanied by more severe long-term behavioral dysfunction and neurological injuries (7). There is evidence indicating that inflammatory cells, such as neutrophils release matrix metalloproteinases-9 (MMP-9) or other MMPs, suggesting that antiinflammatory drugs may decrease MMPs and improve the treatment of brain ischemia (8).

Intravenous alteplase (recombinant tissue plasminogen activator or tPA) is a time-dependent option of reperfusion therapy and the mainstay of acute ischemic stroke treatment, initiating 4.5 hr of symptom onset (9). Intracerebral hemorrhage and reperfusion injury are among the first main complications of thrombolytic therapy (10). Delayed tPA treatment (beyond 4.5 hr post-stroke) is paralleled with complications including hemorrhagic transformation, free radical production, up-regulation of MMP-9, blood-brain barrier (BBB) leakage, and neurovascular cell death (11, 12). Since delayed thrombolysis is a clinical limitation and damages the brain parenchyma, expanding its therapeutic time window may reduce the adverse effects and optimize the treatment. 

Ischemic post-conditioning refers to a series of rapid ischemia and reperfusion cycles in the early phase of reperfusion and is applied by intermittent occlusion/release of bilateral common carotid arteries (13). Ischemic post-conditioning showed cerebroprotection in cerebral ischemia in animal models (14, 15). Ischemic post-conditioning reduces injury to neurons, endothelial cells, and astrocytes and improves BBB integrity (9). Our previous study showed that ischemic post-conditioning decreases the infarct volume or brain edema and ameliorates neurological functions after the embolic stroke model (13). 

In this study, we aimed to investigate the possible neuroprotective effects of post-conditioning after delayed thrombolysis therapy of embolic stroke in female rats. Since the role of this therapeutic strategy has not been investigated in female animals and data are limited, we designed the base of the study of female animals. This study investigated the efficacy of delayed post-conditioning neuroprotection in modulating the infarct volume, brain edema, BBB disintegration, neurological deficits, and MMP-9 levels after delayed thrombolysis in an embolic model of stroke in female rats. 

## Materials and Methods

***Animals and grouping***

For this experiment, female Wistar rats weighing 200–250 g were obtained from the animal house of Rafsanjan University of Medical Sciences. Animals were housed four per cage under standard conditions including a 12 hr light/dark cycle, the average temperature of 20–23 °C, and humidity of 55 ± 2%. Female rats had free access to standard chow and drinking water. Each animal was used only once throughout the study. Experimental procedures were conducted in compliance with recommendations of the institutional Guideline for the Care and Use of Laboratory Animals (NIH Publications No. 8023, revised 1978) and Rafsanjan University Research and Medical Ethics Committees. 

In the first experiment: 32 female rats were randomly divided into vehicle (0.1 ml per 100 g; IV saline), tPA (with the dose of 1 mg/kg; IV), PC, and combination of tPA and PC (embolic stroke was induced for all these groups). tPA or saline was injected after 6 hr and PC was performed after 6.5 hr of stroke. Laser Doppler flowmetry recorded the regional cerebral blood flow (rCBF) from tPA (Actilyse, Boehringer Ingelheim, Germany) starting time or saline injections for 60 min. The infarct volume, brain edema, and behavioral outcomes were evaluated post-stroke in these groups of animals. In the second experiment, animals were divided into the following groups (n=8): vehicle group, tPA group, PC group, and PC + tPA group to evaluate the BBB permeability and MMP-9 level at 48 hr post-stroke. Moreover, vaginal smear monitoring was performed at the beginning of the study. The fresh vaginal smears were prepared and were observed by an optical microscope after air-dryness. Animals possessing the fern leaf pattern of mucus were entered into the study (16, 17).

***The Surgical procedures***

Halothane anesthesia was used for animals. Rats’ body temperature was kept at 37±5 °C during the surgery. Embolus preparation protocol: the femoral artery was catheterized in the donor rats and blood was directly transferred to a PE-50 tube and maintained for 2 hr at 37 °C for clotting and then was refrigerated 22 hr at 4 °C. Then, a two centimeter section of clot-containing tube was separated and the related clot was introduced into a modified tube (with 0.3 mm outer diameter) for injecting in the middle cerebral artery (MCA). Monitoring of rCBF: probe was joined to the right skull bone (1.7 mm posterior and 5 mm mediolateral to bregma) after opening the scalp and separation of the temporalis muscle and thinning the bone with a drill. This measurement started at 5 min before injecting the clot (baseline), at the time of stroke induction, and up to 60 min after injection of tPA (17, 18).

***Embolic stroke induction***

The induction of this model was based on the injection of the clot into MCA via the method of Allahtavakoli (18). In summary, a longitudinal incision (1.5 cm) was made in the ventral cervical skin. Then, the internal carotid artery, right common carotid artery, and external carotid artery were exposed. The distal part of the external carotid artery was ligated and then was cut. The tube containing a clot (20 mm) was fixed to a Hamilton lock syringe and moved 17–19 mm forward in the internal carotid artery until reaching the inside of the MCA for clot injection. The duration of surgery was 30 min/animal.

***Ischemic post-conditioning method***

We induced an ischemic post-conditioning technique 6.5 hr post-stroke. This method was accomplished through occlusion of bilateral common carotid arteries via 4-0 silk strings tying loosely. An optimal pattern was selected for post-conditioning: occluding (10 sec), releasing (10 sec), and repeated for 5 cycles (13). 

***Infarct volume and brain edema evaluation***

Animals were sacrificed 48 hr after induction of the stroke model and their brains were separated. Two-mm-thick coronal sections of the brains were prepared and stained by 2,3,5-triphenyl tetrazolium chloride 2% (Sigma, USA) (for 30 min in 37 °C), then were fixed in formalin 10% solution. Normal tissues were stained red but the infarct parts remained white (unstained). The Image J software (NIH Image, version 1.61) was used for analysis of the infarct zone. The sum of infarct area of all sections was used for calculation of total infarct area and then was multiplied by the section thickness (2 mm) for calculating the infarct volume. 

The wet-dry method was used for brain edema evaluation. Animal brains were separated in the non-injured or injured hemispheres and the wet weight (WW) was recorded. The weights were again recorded after exposure to 100 °C (for 48 hr) for dry weight (DW). The water content percent of each hemisphere (%WC) was calculated through this formula: [1-(DW/WW)]×100. The edema percentage of each brain was determined via subtracting the non-injured hemisphere %WC from the injured hemisphere %WC (17, 19).

***BBB integrity determination***

Blood-brain permeability was evaluated via Evans blue dye. Evans blue dye 2% solution (EB; Sigma, Germany) was administered IV (4 ml/kg) to animals through their femoral vein. One hour after Evans blue dye injection, saline (37 °C) was infused to anesthetized animals through the heart ventricle until obtaining a colorless infusion fluid from the atrium. Then, animals were decapitated and the brains were separated and were weighed. Then, they were fixed in formamide (1 ml/100 mg) (60 °C for 24 hr). The concentration of the dye extracted from each brain was evaluated using spectrophotometry at 620 nm and was reported as absorbance per gram of tissue (20).

***Behavioral analysis***

2-1) The adhesive removal test: for the assessment of sensorimotor function, animals were trained for 3 days before the stroke. They were tested again before surgery and 24 and 48 hr after stroke. The time taken to sense and then remove each adhesive tape strip from the limbs was recorded from the 3 trials (average was reported). 

2-2) Grasping ability and the forelimb strength: for this evaluation, the hanging wire test was used with the following detail; rats were suspended by their forelimbs on a wire, stretched between two posts and 60 cm above a foam pillow. The time (s) to falling was recorded for each animal. The score zero represented falling immediately and the time out period was considered 20 sec. Two trials were performed for every animal and the average was recorded. 

2-3) Neurological deficits: these parameters were recorded 24 hr and 48 hr after stroke. They were determined via a scoring model (Bederson), which are as follows: 0: no observable deficits; 1: the flexion of forelimb; 2: forelimb flexion and a decreased resistance to the lateral push; 3: circling unidirectional; 4: circling unidirectional and decreased levels of consciousness; and 5: death of animal (20).

***MMP-9 serum level assessment***

MMP-9 serum level was determined using an enzyme-linked immunosorbent assay (ELISA) kit (R and D system, USA, Minneapolis) based on the company protocol. Briefly, assay diluents were added to each well, and then serum samples were added to the ELISA plate and incubated for 2 hr at room temperature. Following removing of remaining serum by the wash buffer; rat total MMP-9 conjugate was added and then after 2 hr of incubation was washed. Afterward, the substrate solution was added, and following 30 min, the reaction was stopped using the stop solution. Finally, the optical density was measured using an ELISA reader (USA) at 450 nm (19).

***Statistical analysis***

Data are expressed as mean ± SEM Data were analyzed using GraphPad Prism data analysis program version 6 (GraphPad Software San Diego, CA, USA). One-way analysis of variance (ANOVA) followed by Tukey’s multiple comparisons exhibited the difference within the experimental groups. Two-way ANOVA was used to analyze laser Doppler data. Value<0.05 was defined as statistically significant. 

## Results

***Effects of post-conditioning and tPA on the infarct volume***

**Figure 1:**
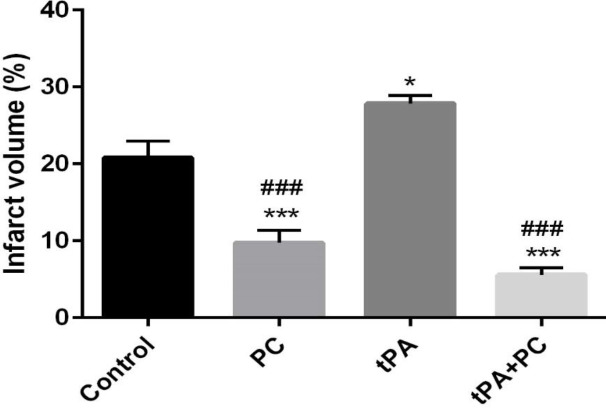
Effects of post-conditioning (PC) and tPA on the infarct volume. Results are illustrated as mean ± SEM. One-way ANOVA and Tukey *post hoc* test were used to compare the values from experimental groups. *P<0.05 and ***P<0.001 vs control, ###*P*<0.001 vs tPA

As shown in Figure 1, PC and tPA+PC groups showed a remarkable reduction in the infarct volume compared with control and tPA groups (*P*<0.001). Meaning that the damage caused by the embolic stroke or tPA therapy was largely neutralized through our experimental manipulation. The tPA group also showed a significant increase in the infarct volume compared with control animals (*P*<0.05).

***Effects of post-conditioning and tPA on brain edema***

**Figure 2:**
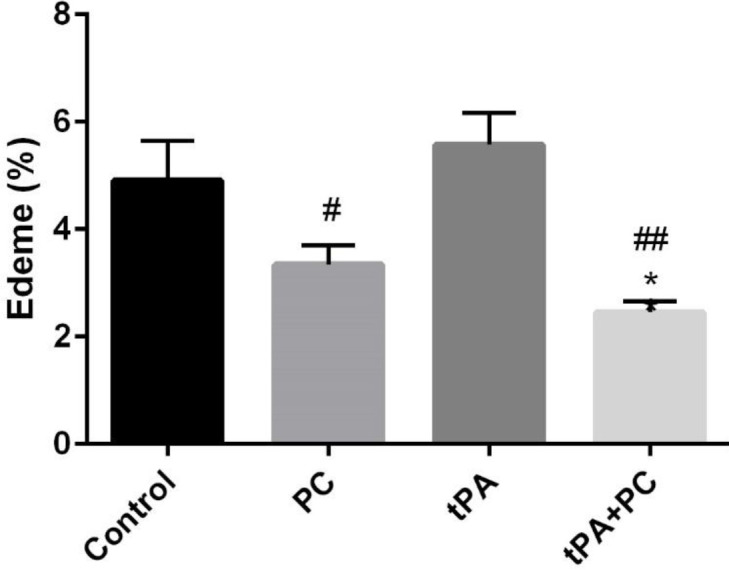
Effects of post-conditioning (PC) and tPA on brain edema. Results are illustrated as mean ± SEM. One-way ANOVA and Tukey *post hoc* test were used to compare the values from experimental groups. *P<0.05 vs control, #*P*<0.05 and ## *P*<0.01 vs tPA

As shown in Figure 2, the tPA+PC group was accompanied by a decrease in brain edema compared with the control group (*P*<0.05). Moreover, both PC and tPA+PC groups showed a significant brain edema reduction compared with the tPA group (*P*<0.05 and *P*<0. 01, respectively). 

***Effects of post-conditioning and tPA on BBB integrity***

**Figure 3:**
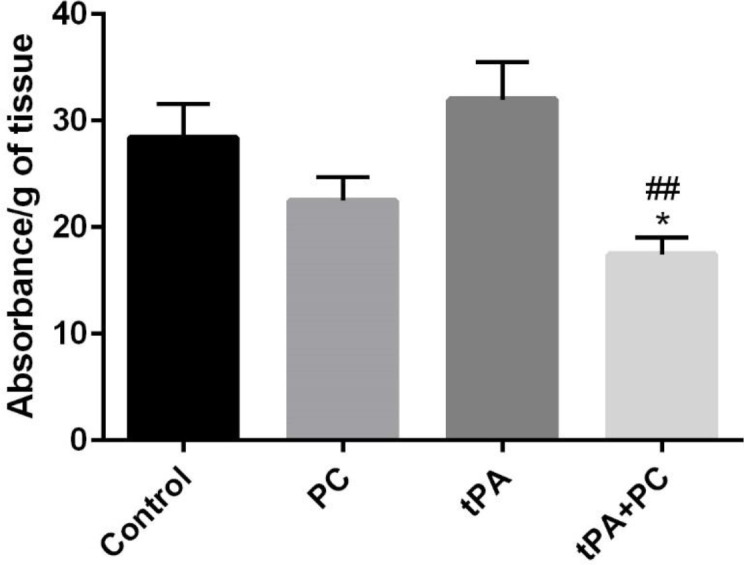
Effects of post-conditioning (PC) and tPA on BBB integrity via Evans blue dye. Results are illustrated as mean ± SEM. One-way ANOVA and Tukey *post hoc* test was used to compare the values from experimental groups. *P<0.05 vs control, ##*P*<0.01 vs tPA

According to Figure 3, tPA+PC group not only showed a significant decrease compared with the control group (*P*<0.05), but also exhibited a significant decrease in comparison with the tPA group (*P*<0.01). This means ability of the tPA+PC group to protect the blood-brain barrier.

***Effects of post-conditioning and tPA on sensorimotor function by adhesive removal test***

**Figure 4:**
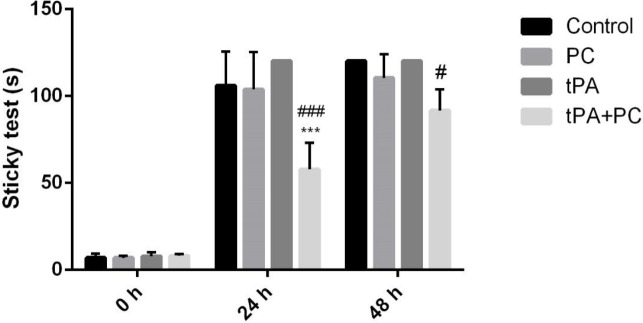
Effects of post-conditioning (PC) and tPA on the sticky test. Results are illustrated as mean ± SEM. One-way ANOVA and Tukey *post hoc *test was used to compare the values from experimental groups. ***P<0.001 vs control and #*P*<0.05 and ###*P*<0.001 vs tPA

As depicted in Figure 4, 24 hr after stroke, the tPA+PC group compared with control or tPA animals, revealed a significant reduction in the time to touch and remove the sticky labels (tPA±PC: 57.62±15.44 vs tPA 119.25±0.75). Furthermore, at 48 hr post-stroke combination of tPA and PC significantly reduced the time for the sticky test in comparison with the tPA group (from around 110 to 82.5) (*P*<0.05). 

***Effects of post-conditioning and tPA on grasping ability and forelimb strength***

**Figure 5:**
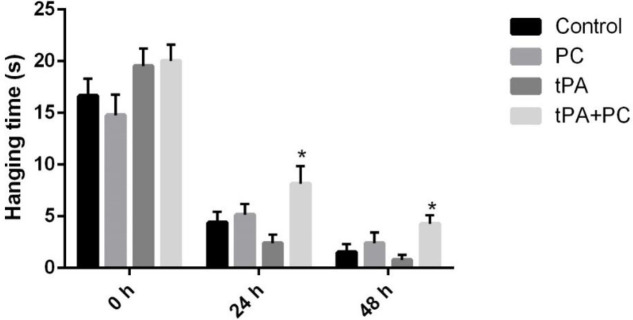
Effects of post-conditioning (PC) and tPA on hanging time. Results are illustrated as mean ± SEM. One-way ANOVA and Tukey *post hoc* test was used to compare the values from experimental groups. *P<0.05 vs tPA

Figure 5, tPA+PC group could improve grasping ability or forelimb strength both 24 and 48 hr after embolic stroke compared with tPA (24 hr: 8.12±1.70 vs 2.37±0.84; *P*<0.05) (48 hr: 5.16±0.74; *P*<0.05). PC group could enhance the hanging time in comparison with control or tPA at both 24 and 48 hr but these levels were not significant. 

***Effects of post-conditioning and tPA on neurological deficits by Bederson’s test***

**Figure 6:**
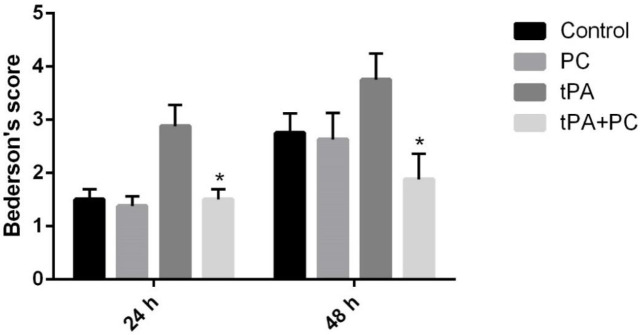
Effects of post-conditioning (PC) and tPA on neurological deficits. Results are illustrated as mean ± SEM. One-way ANOVA and Tukey *post hoc *test was used to compare the values from experimental groups. *P<0.05 vs tPA

Figure 6 shows the results obtained from the neurological damage assessment. Based on this figure; 24 and 48 hr after stroke, a significant decrease in Bederson’s score was observed in the tPA+PC group compared with the tPA group, displaying an improvement in behavioral function (*P*<0.05). 

***Effects of post-conditioning and tPA on MMP-9 serum level***

**Figure 7:**
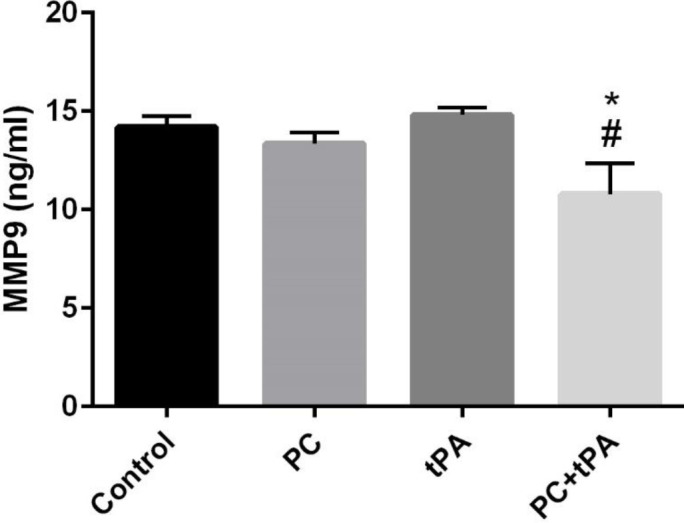
Effects of post-conditioning (PC) and tPA on MMP-9 serum level. Results are illustrated as mean ± SEM. One-way ANOVA and Tukey* post hoc* test was used to compare the values from experimental groups. *P<0.05 vs control. #*P*<0.05 vs tPA

Figure 7 shows that the tPA+PC group significantly diminished the serum level of MMP-9 compared with control or tPA groups (both *P*<0.05); displaying that tissue degradation or inflammation was reduced in this group.

***Effects of post-conditioning and tPA on the MCA region blood flow***

**Figure 8:**
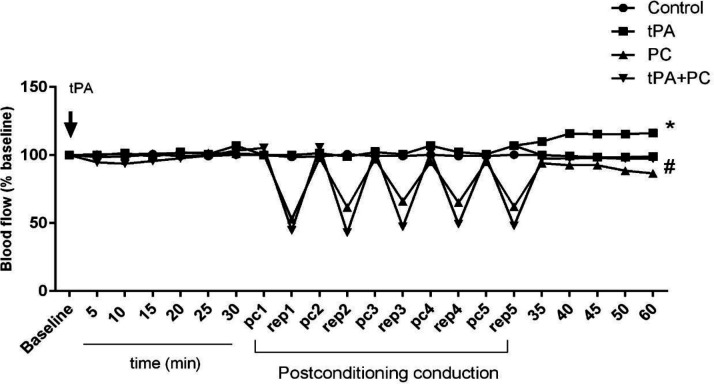
Effects of post-conditioning (PC) and tPA on blood flow of MCA region. Results are illustrated as mean ± SEM. Two-way ANOVA was used to compare the values obtained from different experimental groups. *P<0.05 vs control #*P*<0.05 vs tPA

Figure 8 shows that tPA from 35 min after administration significantly rises the blood flow compared with control (*P*<0.05). Application of PC after 30 min of tPA administration inhibited that remarkable enhancement of blood flow compared with tPA (*P*<0.05). 

## Discussion

This study was designed to investigate the effect of post-conditioning on the stroke consequences after administration of late tPA in female animals. This study shows that application of delayed PC has resulted in reduction of the infarct volume. PC application with tPA therapy prevented the intense enhancement in CBF. PC combination with tPA led to a significant fall in the infarct volume, brain edema, BBB destruction, behavioral damage, and MMP-9 serum level. Our results indicate that delayed post-conditioning is neuroprotective in combination with delayed tPA after embolic stroke in the female animals. These protective effects may be mediated via reduction of reperfusion injury or other mechanisms discussed below.

It is well documented that sex differences existed in experimental stroke consequences. These differences include larger infarct volume, greater stroke-induced mortality, more severe sensory-motor dysfunctions, and higher BBB permeability in adult males (21). Moreover, there is ample evidence that stroke pathophysiology is sex-specific and this point necessitates the consideration of sex in the development of pharmacological approaches in prevention or treatment of stroke (22, 23). Bushnell *et al*. in their study suggested that developing safe and efficacious neuroprotective strategies with sex-specificity is a pivotal factor in decreasing the morbidity and mortality of stroke (22). Bonkhoff *et al*. revealed that acute ischemic stroke outcomes are related to the sex-specific lesion patterns and suggested that sex-stratified approaches in acute ischemic stroke management should be investigated (24). A study investigated sex differences in ischemic stroke patients treated with tPA (25). In another study, researchers showed sex differences in transient ischemic attack at presenting symptoms, causes and outcomes and encouraged further studies which take into account these differences for prevention and treatment (26). A study investigated the estrogen levels in postmenopausal women with ischemic stroke and showed that this level was lower than the control group or normal estrogen level in the healthy menopausal women (27). Moreover, a study revealed that selective activation of estrogen receptor β alleviates ischemic neuronal injury and inhibits stroke neuroinflammatory damage without oncogenic events of estrogen and may be a therapeutic strategy in postmenopausal women (28). In line with the mentioned studies, the current study was designed with female animals for observation of a therapeutic approach. 

Ischemic postconditioning (PC) showed neuroprotective effects against transient or permanent models of stroke in both *in vivo* and *in vitro* studies (29, 30). Esmaeeli-Nadimi *et al*. in their study on an embolic stroke model revealed that PC conduction with 5 cycles including 10 sec occlusion (10 sec) and reperfusion (30 sec) of bilateral common carotid arteries at 6.5 hr after stroke reduces BBB disruption, edema of the brain, neurological dysfunctions, and peri-infarct apoptosis in male rats. They showed that PC reduced the reperfusion injury after delayed tPA thrombolysis and widened the therapeutic time window of tPA in male rats (17). As noted above, the investigation of sex differences in stroke studies is suggested previously. In the current study, we showed that PC application at 6.5 hr post-stroke in female animals could affect the infarct volume. Meaning that delayed PC could display neuroprotection in female animals same as for male animals. Furthermore, delayed PC conduction with tPA therapy, lowered CBF, infarct volume, brain edema, and BBB destruction in female stroke animals in this study. Literature review showed that estrogens such as 17β-estradiol could attenuate BBB disruption induced through transient cerebral ischemia in female rats. Estrogens also could modulate the activity of MMPs such as MMP-2 and MMP-9 in ovariectomized rats under cerebral ischemia-reperfusion injury (31). Our study showed that in the PC+tPA group of female animals the level of MMP-9 was lower than control or PC alone. This protective effect may be to some extent related to the role of estrogens. Besides, a study revealed that intranasal 17β-estradiol loaded gelatin nanoparticles conferred neuroprotection in the post-ischemic brain of adult male mice through reduction of infarct volume (32). Further studies are needed in the future to elucidate the effects of PC in female stroke models treated with tPA; including the expression of estrogen receptors and estrogen level assessment especially in aged female animals. In our study, the PC+tPA group showed reduction of neurobehavioral deficits which is paralleled to Esmaeeli-Nadimi *et al*. study for delayed PC and tPA effects on stroke model. As we study these behavioral changes in female animals we can point to other studies exhibiting that estradiol may modify synaptic transmission, post-stroke neurogenesis and also improve behavioral dysfunctions (33). By the abovementioned evidence, conduction of delayed PC in female rats with delayed tPA treatment could be an effective strategy for extending the time window of tPA and attenuation of stroke damage, and the future translation for female patients could be interesting. Another mechanism for the protective role of PC on female animals in this study could be attributed to reduction of post-ischemia inflammation, because, we observed a diminished level of MMP-9 in the PC+tPA group which may be an indicator of inflammation. In this regard, Chen *et al*. in their study investigated a sex-specific neuroprotective strategy against stroke which was used delayed at 48 hr following reperfusion and could inhibit neuro-inflammation and reduce the level of MMP-9 and inducible nitric oxide (34). 

## Conclusion

In this study, the effects of PC were assessed with delayed tPA therapy in female rats. Our data supported that delayed PC and tPA are neuroprotective, broadening the time window of tPA in female rats under embolic stroke. 

## Authors’ Contributions

MM (first author) and MM (second author) performed the experimental procedure and handled animals throughout the study. FM had a role in preparing the revised version. MA had the main idea and managed the study stages. AK, JH, and AS had roles in statistical analysis and organizing the main materials and manuscript revisions. EH had a role in manuscript preparation. MH had a role in study design and manuscript preparation. All authors have read and approved the final version of the manuscript 

## Conflicts of Interest

There are no conflicts of interest.
